# Reactive Cellu-mers—A Novel Approach to Improved Cellulose/Polymer Composites

**DOI:** 10.3390/polym14091670

**Published:** 2022-04-20

**Authors:** Dariya Getya, Ivan Gitsov

**Affiliations:** 1 Department of Chemistry, State University of New York—ESF, Syracuse, NY 13210, USA; dgetya@syr.edu; 2 The Michael M. Szwarc Polymer Research Institute, Syracuse, NY 13210, USA; 3 The BioInspired Institute, Syracuse University, Syracuse, NY 13244, USA

**Keywords:** copolymerization, cellulose nanofibrils, polystyrene, semi-interpenetrating networks, polymer nanocomposites

## Abstract

In this paper, we describe a novel method for preparation of polymer composites with homogeneous dispersion of natural fibers in the polymer matrix. In our approach, Williamson ether synthesis is used to chemically modify cellulose with polymerizable styrene moieties and transform it into a novel multifunctional cellu-mer that can be further crosslinked by copolymerization with styrene. Reactions with model compounds (cellobiose and cellotriose) successfully confirm the viability of the new strategy. The same approach is used to transform commercially available cellulose nanofibrils (CNFs) of various sizes: Sigmacell and Technocell™ 40, 90 and 150. The styrene-functionalized cellulose oligomers and CNFs are then mixed with styrene and copolymerized in bulk at 65 °C with 2,2′-azobisisobutyronitrile as initiator. The resulting composites are in a form of semi-interpenetrating networks (s-IPN), where poly(styrene) chains are either crosslinked with the uniformly dispersed cellulosic component or entangled through the network. Non-crosslinked poly(styrene) (31–41 w%) is extracted with CHCl_3_ and analyzed by size-exclusion chromatography to estimate the extent of homopolymerization and reveal the mechanism of the whole process. Electron microscopy analyses of the networks show the lack of cellu-mer agglomeration throughout the polymer matrix. The homogeneous distribution of cellulose entities leads to improved thermal and mechanical properties of the poly(styrene) composites compared to the physical mixtures of the same components and linear poly(styrene) of similar molecular mass.

## 1. Introduction

Polymer composites are playing an ever-increasing role in a broad array of technologies and advanced applications from automotive and aerospace industries [1] to civil engineering [2]. Various substances are employed as fillers in these composites, clay being one of the most common [3]. Other naturally derived additives are also actively investigated including wood [4] and cellulose, the most abundant plant biomass polymer [5]. Cellulose is not only a biodegradable, biocompatible and renewable low-cost material, but also has many attractive characteristics—low density, high specific surface area, high elastic modulus and low coefficient of thermal expansion [6]. Addition of cellulose nanocrystals (CNCs) or nanofibers (CNFs) to a polymer matrix has been proven to enhance the mechanical and thermal properties of the resulting nanocomposites [7,8,9] and networks [10]. The positive effect of these natural nanofillers is due to their higher surface area to volume ratio, ensuring an enhanced surface contact with the matrix polymer [11,12]. That is why the interest towards cellulose reinforced nanocomposites is increasing. Applications of such materials include but are not limited to the paper and packaging industry [13], electronics [14], composites and fillers [15] and cosmetics [16,17]. A significant limiting factor for the use of cellulose as a nanofiller is the hydrophilic character of its surface. The direct incorporation of CNCs or CNFs into hydrophobic thermoplastic matrixes has some negative effects on the mechanical properties of resulting nanocomposites due to the formation of agglomerates during the dispersion process [18]. Commonly polymer and cellulose mixtures must be heated at first to the melting temperature of the polymer, and then the compression molded to products. Most of thermoplastic polymers are processed at higher temperatures, and cellulose fibers undergo thermal degradation. Moreover, the mechanical properties of the composite may be highly anisotropic, causing a directional dependence of the tensile modulus [19].

Chemical modification of the CNC/CNF surface is a successful strategy to overcome the incompatibility limitation [20,21,22]. It alleviates several problems, with the phase segregation being one of the most difficult to overcome. The incorporation of specific surface moieties improves the miscibility between components, allowing for modified cellulose to be more evenly blended with the polymer “host”. The net result is the formation of a polymeric material with improved mechanical properties.

The chemical surface modification of cellulose is performed by selective grafting. The essential task here is to preserve the fibrillar structure of the cellulose chain, but at the same time to incorporate new fragments [23]. There are two main approaches to how a polymer chain could be attached to the cellulose backbone: ‘‘Grafting-to’’ and ‘‘grafting-from’’.

These approaches are schematically represented in Figure S1. In the ‘‘grafting-to’’ method, pre-made polymer chains with their reactive chain ends are directly attached onto the cellulose backbone [24]. In the ‘‘grafting-from’’ approach, polymer grows from reactive sites that are formed on cellulose chain.

The most widely used approach is ‘‘grafting-from’’. It enables high graft density due to easy monomer access to the reactive surface groups. However, the polymers formed must be somehow cleaved from the backbone to be further analyzed [25]. The ‘‘grafting-to’’ approach is inconvenient because of steric hindrances between components. Here, slow and uneven diffusion of chain ends to the cellulose surface groups complicates attachment due to spatial confinement and leads to low coverage and non-uniformity of the surface. The advantage of this strategy is that the molecular mass characteristics of the polymers to be grafted can be easily controlled and manipulated before being coupled [26].

All the above-mentioned methods, however, produce surface-modified CNCs or CNFs that still must be mixed with a polymer matrix in order to create a polymer nanocomposite. This process is still plagued by potentially random distribution of the reinforcing agents within the polymeric matrix.

**Scheme 1:**
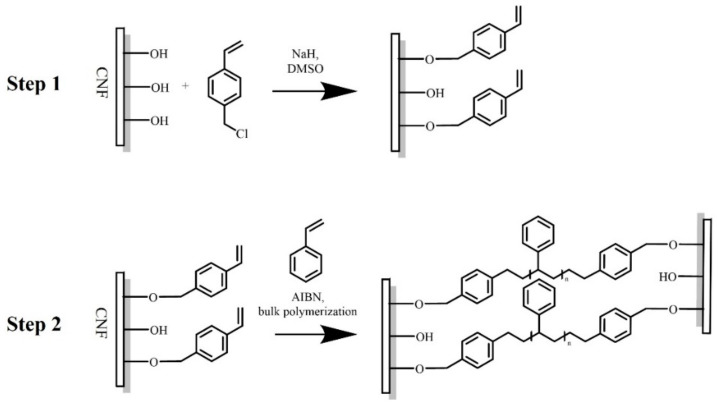
Surface modification of CNF and subsequent copolymerization with St.

**Figure 1:**
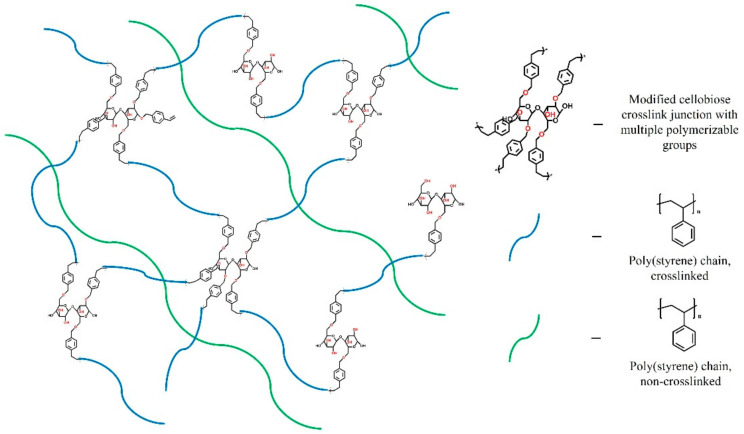
Schematic representation of PSt semi-interpenetrating network formed by copolymerization of modified cellobiose and St.

In this paper we describe a novel alternative approach to cellulose-based composites. It is still based on the “grafting from” strategy, but instead of growing the whole polymer chain on the cellulose surface, multiple monomer moieties are surface-attached, forming multi-functional cellulosic macromonomers—cellu-mers—which are then copolymerized. Our hypothesis is that the copolymerization mixture will be homogeneous, avoiding in this way cellulose agglomeration. As a proof of principle, cellobiose and cellotriose that serve as low molecular mass analogues for CNFs are surface-modified with styrene (St) moieties by Williamson ether synthesis (Scheme 1, step 1). This process facilitates the direct incorporation of polymerizable units onto the cellulose, repeating units in one step. The resulting reactive cellu-mers are then copolymerized with St using classic radical polymerization, Scheme 1, step 2. The potential benefits of this new strategy are that the styrenic cellu-mers act not only as comonomers, but also serve as reinforcing fillers and are covalently bound throughout the poly(styrene), PSt matrix forming a semi-interpenetrating network (semi-IPN) without phase separation, Figure 1. Semi-IPNs that are formed using this method would have cellu-mers evenly distributed throughout the PSt composite without further optimization of the crosslinking protocol.

## 2. Materials and Methods

### 2.1. Materials

Sigmacell cellulose (Type 101, Sigma-Aldrich, 3050 Spruce St., St. Louis, MO, USA) and Technocell™ cellulose fibers, Technocell 40 (T-40), Technocell 90 (T-90) and Technocell 150 (T-150) from Cellulose Filler Factory Corporation (10200 Worton Rd, Chestertown, MD, USA) were used as received. St (≥99%), 4-vinylbenzyl chloride (4-VBC, 90%), 2,2′-azobisisobutyronitrile (AIBN, 98%) were supplied by Millipore Sigma (3050 Spruce St., St. Louis, MO, USA). St was used after inhibitor removal by activated alumina, Al_2_O_3_ (activity grade I, ICN Biomedicals, 3300 Hyland Ave., Costa Mesa, CA, USA). D-(+)-cellobiose and cellotriose were purchased from TCI America (9211 N Harborgate St., Portland, OR, USA) and were used without further purification. Dimethyl sulfoxide (DMSO, EMD Millipore Corporation, 290 Concord Rd., Billerica, MA, USA), chloroform (J. T. Baker, 222 Red School Ln. Phillipsburg, NJ, USA) and methanol (Burdick & Jackson, 1953 Harvey St., Muskegon, MI, USA) were used as received. 

### 2.2. Methods

#### Preparation of Reactive Cellu-Mers Based on Cellobiose, Cellotriose or Cellulose Nanofibers (CNFs)

Firstly, 0.2 g of а starting compound (cellobiose, cellotriose, cellulose CNF) was dissolved or dispersed (depending on the compound) in 3 mL anhydrous DMSO. A predetermined amount of the 4-VBC (1–30 eq) was then added. After stirring the mixture for 5 min, NaH is added into the reaction system and kept at room temperature (RT) for 24 h. After that time, MeOH was added to quench the reaction and the reaction mixture was vacuum-filtered and washed with ethanol. The unreacted 4-VBC was removed by extraction with hexanes. The modified cellobiose (cellobiose-m) was obtained as a pale, yellow-colored liquid; cellotriose-m and CNFs-m were obtained as pale, yellow-colored solids. The yields were as follows: Cellobiose-m 84% and cellotriose-m 72%. CNFs contained up to 91 mg of added methylstyrene fragment in 1 g of the CNF. The degree of substitution varied with the number of equivalents of 4-VBC added to the reaction mixture.

### 2.3. Characterization of Cellobiose-m, Cellotriose-m and CNF-m

#### 2.3.1. Matrix Assisted Laser Desorption/Ionization Time-of-Flight Mass Spectrometry (MALDI-TOF MS)

Analyses were performed on a MALDI TOF/TOF mass spectrometer, Bruker AutoFlex III (Bruker Corporation, Billerica, MA, USA) equipped with a Smartbeam II laser source (Nd:YAG laser, 266 or 355 nm). Using 2,5-dihydroxybenzoic acid (DHB) as a matrix. Matrix solution (30 mg/mL) and sample solution (3 mg/mL) were mixed in a 1:1 ratio. Samples were spotted on an MTP 384 target plate (polished steel, Bruker Daltonics, Billerica, MA, USA). Spectra were recorded in the range 200–3000 Da (linear positive mode). Knowing the concentration of samples, peak *m/z* values and intensities from MALDI analysis, the number average molecular mass Mn and weight average molecular mass Mw were calculated using Equations (1) and (2), respectively (N_i_—mole fraction of chains with molecular mass M_i_).

(1) $$\mathrm{Mn}=\frac{\sum N_{i}M_{i}}{\sum N_{i}}$$

(2) $$\mathrm{Mw}=\frac{\sum N_{i}M_{i}{}^{2}}{\sum N_{i}M_{i}}$$

#### 2.3.2. Fourier Transform Infrared (FT-IR) Analysis

The analysis was conducted on a Bruker Tensor 27 IR (Bruker Corporation). The instrument was equipped with a mid-infrared (MIR) source and a Deuterated Lanthanum α Alanine doped TriGlycine Sulphate (DLaTGS) detector. Spectra were recorded in the range 4000–600 cm^−1^ under ambient conditions at a resolution of 4 cm^−1^. A total of 48 scans were collected for each spectrum in addition to the background.

#### 2.3.3. Ultraviolet-Visible (UV-VIS) Spectroscopy

The degree of substitution (DS) was estimated using Agilent 8453 UV-Visible spectrophotometer (Agilent Technologies, Santa Clara, CA, USA). First, a calibration curve was built using 4-VBC solutions at various concentrations in DMSO, Figure S2. Then, the synthesized products were analyzed with pure DMSO as a blank solution.

### 2.4. Synthesis of Semi-IPN Containing Cellobiose-m, Cellotriose-m or CNFs-m, and PSt 

A necessary amount of crosslinker (cellobiose-m, cellotriose-m abnd CNFs-m), St and AIBN (1:100:0.5 weight ratio) was placed into a round bottom flask equipped with a magnetic stirrer. Such a weight ratio was determined experimentally as the most yield-effective for semi-IPN synthesis. The bulk polymerization was conducted at 65 °C under an argon atmosphere for 6 h. After that time, the solidified reaction mixture was extracted with chloroform and further analyzed. The amount of the extracted homo PSt was determined gravimetrically, and its molecular mass characteristics were defined by size-exclusion chromatography.

### 2.5. Characterization of CNFs and PSt Semi-IPNs

#### 2.5.1. Scanning Electron Microscopy (SEM)

The surface morphology of swollen extracted IPNs was examined by a JSM 5800LV scanning electron microscope (JEOL, Tokyo, Japan). Samples were cryo-fractured and palladium-coated under vacuum before the electron micrographs were recorded.

#### 2.5.2. Transmission Electron Microscopy (TEM)

A block of semi-IPN was sectioned using Ultramicrotome Leica EM UC6 (Leica Microsystems, Buffalo Grove, IL, USA). The grid was vacuum-dried for 24 h and then stained with 2% uranyl acetate. Grids were then analyzed by TEM with a JEOL JEM-2100F microscope at an accelerating voltage of 200 kV. The images were recorded by Gatan OneView CCD camera (Gatan-Ametek, Plesanton, CA, USA).

#### 2.5.3. Size-Exclusion Chromatography (SEC)

The analyses of the extracted homo PSt were performed on a system with an M510 pump, U6K universal injector, 486 tunable absorbance detector (all from Waters Corporation, Milford, MA, USA) and 250 dual refractometer/viscometer detector (Viscotek/Malvern Corporation). The separation was achieved over a set of three 5 µm Styragel columns (HR 2, 3 abnd 5, Waters Corporation) and calibrated with 17 narrow dispersity PSt standards with molecular masses between 0.162 kDa and 956 kDa.

#### 2.5.4. Swelling Studies

Swelling behavior was studied by a gravimetric method. In this case, a dry network sample with known weight was immersed in chloroform at room temperature (RT). After defined periods of time, the swollen gel was weighted, and the swelling degree (SD) was calculated using the following Equation (3):

(3) $$\mathrm{SD}\;(\%)=[\left(W_{s}-W_{0})/W_{0}\right]\;\text{×}\;100,$$

where $W_{s}$ is the weight of the swollen gel at time t and $W_{0}$ is the weight of the dry gel.

#### 2.5.5. Differential Scanning Calorimetry (DSC)

Glass transition temperatures (T_g_) and heat capacity jump (ΔC_p_) at the glass transition were measured using a differential scanning calorimeter DSC Q200 (TA Instruments). Samples were analyzed in heating–cooling–heating cycles from 0 to 180 °C at a scanning rate of 10 °C/min using dry nitrogen flow. The second heating cycle was used to determine the T_g_ of each nanocomposite.

#### 2.5.6. Dynamic Mechanical Analysis (DMA)

DMA was performed using a TA Q800 analyzer (TA Instruments, New Castle, DE, USA) with DMA Multi-strain module type and single cantilever clamp in a temperature range of 0–150 °C at a heating rate of 1 °C/min and a frequency of 5 Hz. Specimens with typical dimensions 25/12/2 mm (l/w/th) were prepared via thermal bulk radical copolymerization using silicone molds.

## 3. Results and Discussion

### 3.1. Synthesis of St-Modified Cellobiose and Cellotriose

**Scheme 2:**
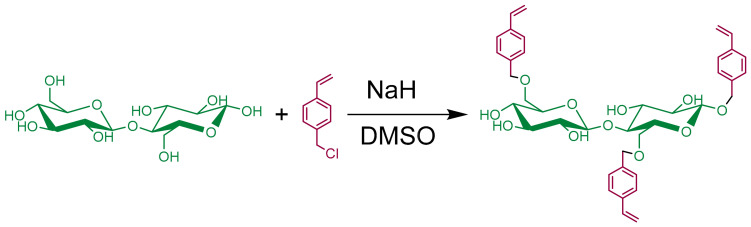
Tentative modification of cellobiose via Williamson ether synthesis.

**Figure 2:**
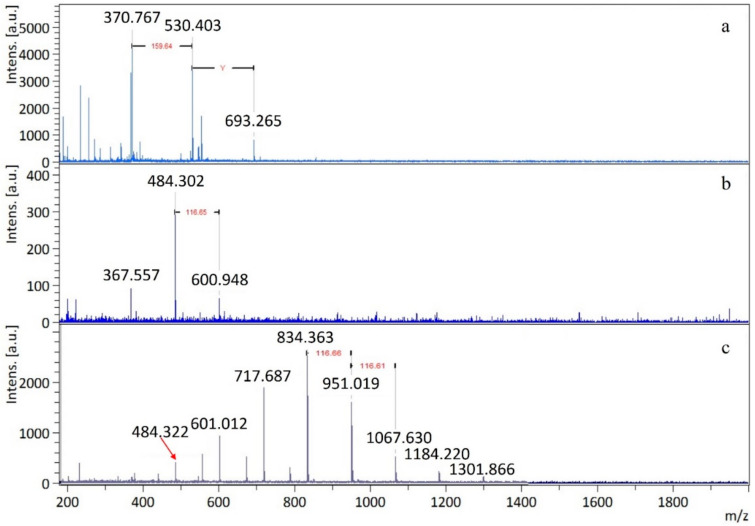
MALDI-TOF spectra of cellobiose modified with eight equivalents of 4-VBC at 25 °C. (**a**) neat cellobiose; (**b**) modified cellobiose after 5 h; (**c**) modified cellobiose after 22 h. DHB was used as a matrix.

The multi-functional cellu-mers were successfully synthesized via Williamson ether synthesis. Cellobiose and cellotriose (cellulosic disaccharide and trisaccharide) served as model compounds, as they have the same repeating units, but smaller molecular mass and are easier to characterize. The cellobiose modification is shown in Scheme 2. This oligomer contains eight hydroxyl groups and each of those can participate in the etherification reaction. For simplicity, Scheme 2 shows a reaction product where only the primary hydroxyls are substituted; the real degree of substitution depends on the amount of 4-VBC used. The extent of cellobiose modification was monitored by MALDI-TOF analysis and is shown on Figure 2. Cellobiose sodium ion adduct of [M+Na]^+^ is observed at 370 *m/z*, while signals at 530 and 693 *m/z* correspond to cellotriose and cellotetraose impurities that are present in the starting compound. Each of them differs from the previous one by 160 *m/z,* which corresponds to one anhydroglucose unit (Figure 2a) [27,28,29]. After 5 h of the reaction, new signals appear at 484 and 600 *m/z*, indicating that one and/or two cellobiose -OH groups are now substituted by methylstyrene moieties (Figure 2b). These peaks differ by 117 *m/z*, which corresponds to one methylstyrene group that is added to the structure. Lastly, Figure 2c shows the modified cellobiose mixture after 22 h, where several higher substituted products appear with a small fraction of fully substituted cellobiose also present (peak at 1301.866 *m/z*). 

In order to control the degree of cellobiose substitution, the modification reaction was conducted with various amounts of the 4-VBC: 1, 2, 4, 6, 8 and 30 eq. All the other reaction conditions (temperature, time, solvent) were kept the same. Figure S3 contains the MALDI-TOF spectra of the reaction products that were obtained. In cases when 1 and 2 eq were added (Figure S3a,b) the substitution occurred only in 2-OH groups. These are probably the most reactive primary hydroxyls. When more equivalents of 4-VBC were added, the degrees of substitution increased, with 5.09 being the maximum average degree of substitution (DS).

Cellobiose is soluble in DMSO at elevated temperatures, so 4-VBC can be added either immediately after the dissolution while the solution is still hot (~100 °C), or when the solution is cooled down to room temperature. Figure S4 and Table 1 show how the average molecular mass, and DS depend on the temperature, at which 4-VBC was added. Both reaction mixtures contain the same modified oligomers, but their amounts differ, affecting the average molecular masses, Table 1. It should be mentioned that the 100 °C experiment cannot be conducted for extended periods of time because the reaction between NaH and DMSO is exothermic and poses a safety risk [30].

**Figure 3:**
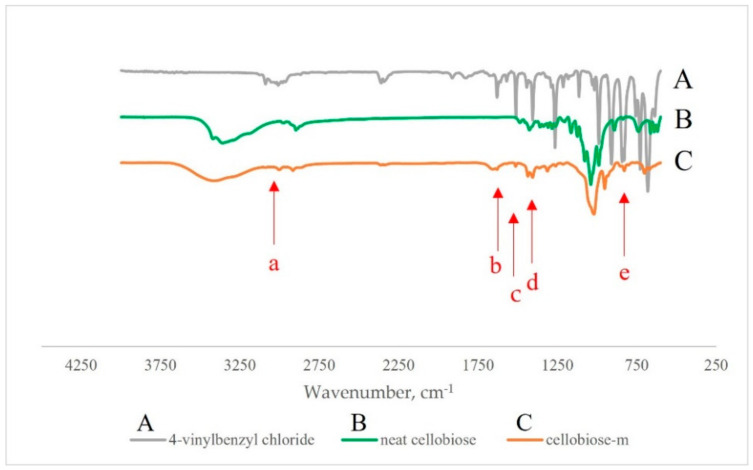
IR spectra of cellobiose before and after the 4-VBC modification: (**A**) 4-VBC; (**B**) neat cellobiose; (**C**) cellobiose modified with six equivalents of 4-VBC (cellobiose-m).

After the removal of unreacted 4-VBC, new absorption bands were observed in the FT-IR spectra of all cellobiose-m samples, Figure 3. The C–H aromatic stretch vibration appeared around 3000 cm^−1^ (Figure 3C(a)) and the C–H out-of-plane vibration was visible around 830 cm^−1^ (Figure 3C(e)). The C=C alkene band at 1600 cm^−1^ (Figure 3C(b)) and C=C aromatic bands showed at 1550 and 1475 cm^−1^ (Figure 3C(c,d)) along with the carbohydrate –C–O–C– vibration at 1120 cm^−1^ [31,32]. These bands confirm the incorporation of methylstyrene moieties into the cellobiose structure.

Since cellobiose is UV-transparent, the presence of absorbance in this spectral region could be directly related to DS and could be quantified with a concentration calibration curve made with 4-VBC (Figure S2). With the increase in the 4-VBC amount used for the modification, the UV absorbance of cellobiose-m increases and shifts to a longer wavelength—a clear indication that more St units were attached to the carbohydrate (Figure S5). DS derived from the calibration with 4-VBC and recorded spectra is shown in Figure S6. It is seen that even a dramatic increase in the 4-VBC/cellobiose ratio did not result in full substitution of all eight hydroxyl groups (DS~5.09). While the mass spectra (Figure S3d–f) confirm the presence of fully substituted cellobiose molecules, their content is rather low and therefore the lower-molecular mass species contribute more to the average value. 

After the successful modification of cellobiose (a disaccharide), a modification of cellotriose (a trisaccharide) was conducted, as well. This molecule contains one more anhydroglucose unit in its structure and has 11 hydroxyl groups in total. Modification reaction was conducted using 24 eq of 4-VBC and all the other reaction conditions were the same as in cellobiose modification. Figure S7a shows the MALDI-TOF spectrum of net cellotriose (the structure is depicted in the upper right corner). Cellotriose is much more soluble in DMSO than the cellobiose and, most likely, is more prone to react under these conditions. Remarkably, the modification of cellotriose formed only three products, as revealed by the MALDI-TOF analysis. The sodium ion adduct [M+Na]^+^ is observed at 530 *m/z* (Figure S7a) and a potassium ion adduct [M + K]^+^ at 543 *m/z* (Figure S7b). The signal at ~365 *m/z* in the figure is the sodium ion adduct of cellobiose [M+Na]^+^ present in the commercial cellotriose as an impurity [29,33,34]. When reaction time reached 16 h (Figure S7b), cellobiose and cellotriose still existed in the reaction mixture (at 365 and 543 *m/z*, respectively). The cellotriose potassium ion adduct disappeared after 24 h of reaction. The peak at 788 *m/z* corresponds to the sodium ion adduct of a reaction product, with three OH groups substituted. The peak at 1301 *m/z* represents a product with seven substituted OH groups and lastly, the peak at 1814 *m/z* could be assigned to the cellotriose with all 11 hydroxyl groups modified. The calculated average degree of substitution DS is 3.03 by calibrated UV-Vis spectroscopy (Figure S8). As it was in the case of cellobiose-m, the calculated value of 3.03 is an average DS.

### 3.2. Synthesis of St-Modified Cellulose Nanofiber CNFs

Successful modification of both cellobiose and cellotriose proved that our modification strategy works on oligosaccharides of different sizes. Our next step was to modify cellulose nanofibers (CNFs). The tested types of cellulose fibers: Sigmacell and Technocell™ varied in their particle size distribution, ranging from 20 μm (Sigmacell) up to 90 μm (Technocell 90 and 150), Table 2.

Modification reactions were conducted using the same procedure as in the modification of oligosaccharides. The only difference was that CNFs were not dissolved in DMSO, but dispersed, so the modification reaction practically occurred on the surface of the fibers only, leading to the modification of hydroxyl groups that were available at the surface.

The DS dependence on the amount of 4-VBC used for the modification reaction was investigated with Sigmacell CNFs using four different 4-VBC molar equivalents: 3, 8, 15 and 30 eq. The amount of 4-VBC to be added was chosen by calculating the number of repeating units in a certain mass of cellulose. For example, 3 eq of 4-VBC means that in the reaction mixture there are three molecules of 4-VBC per one cellulose hydroxyl group. 

**Figure 4:**
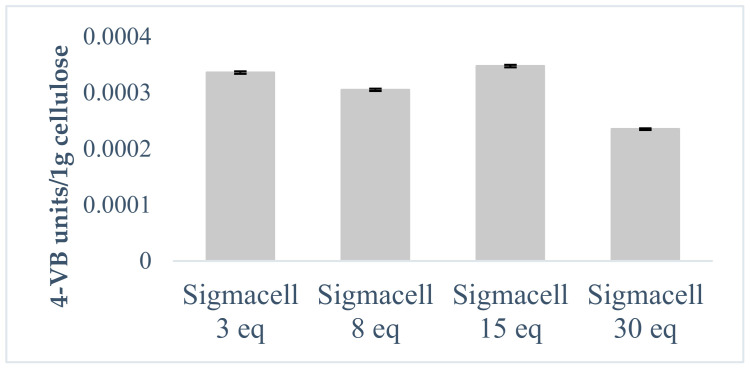
Number of 4-VBC groups attached to 1 g of Sigmacell depending on equivalents of 4-VBC used. Calculated by measuring the UV absorption of DMSO suspensions at 265 nm.

Figure 4 shows the calculated number of 4-VBC units attached to 1 g of cellulose. The data reveal that with the increase in the amount of 4-VBC added, the number of the attached St units does not change significantly and stays around 0.0003 VB groups per 1 g of cellulose. If one considers the number of repeating units in this 1 g (0.0013698) then it turns out that on average each fourth or fifth repeating unit contains a methylstyrene moiety. It should be emphasized that these numbers are underestimated because the UV analysis was performed on suspensions. It is also worth noting that attached polymerizable groups are probably quite randomly distributed on the surface. 

The modification of the Technocell series was conducted using eight equivalents of 4-VBC with similar substitution efficiency, Figure S9.

**Figure 5:**
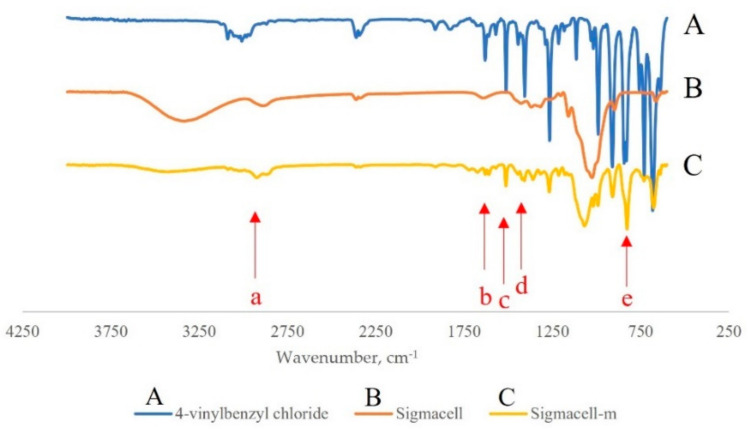
IR spectra of cellulose modification reaction: (**A**) 4-VBC; (**B**) neat Sigmacell; (**C**) Sigmacell-m.

The IR spectrum of Sigmacell cellulose modified with eight equivalents of 4-VBC is shown in Figure 5. The sample was washed with methanol and chloroform prior to the analysis to ensure the removal of residual unreacted 4-VBC. The broad stretching and banding vibration of the cellulose OH groups around 3400 cm^−1^ was visibly diminished (Figure 5B,C). The C-H aromatics stretch vibration at around 3000 cm^−1^ appeared next to the asymmetric C-H stretching of the cellulose ring (Figure 5C(a)). An aromatic C-H out-of-plane vibration at around 830 cm^−1^ (Figure 5C(e)) was also visible. C=C aromatic bands showed at 1550 and 1475 cm^−1^ (Figure 5C(c,d)) and C=C alkene band at 1600 cm^−1^ (Figure 5C(b)) [31,32]. All these new bands confirm the incorporation of St units into the CNF structure.

**Figure 6:**
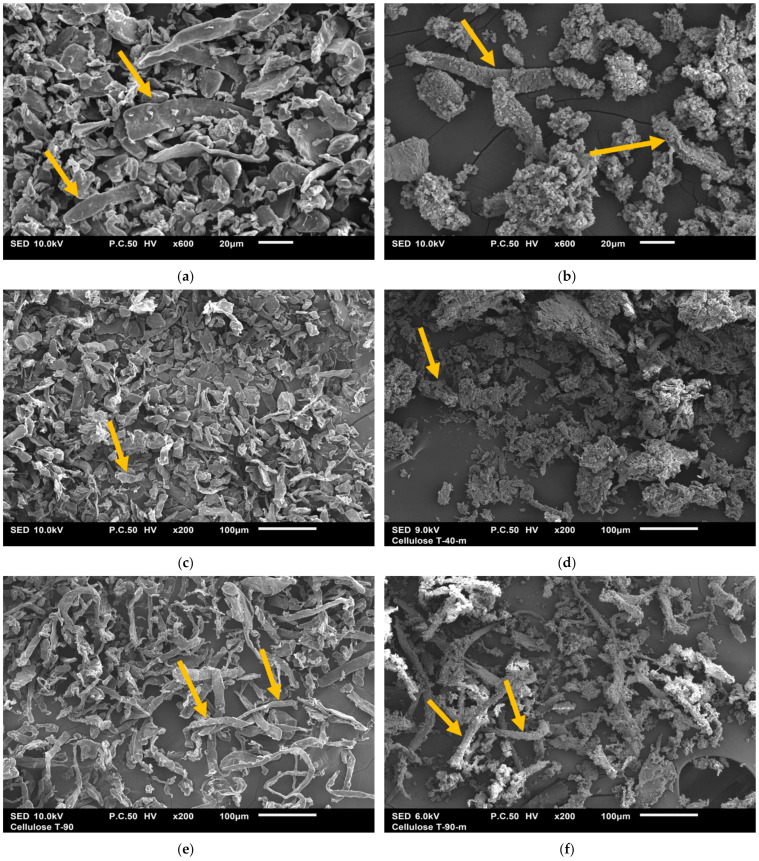

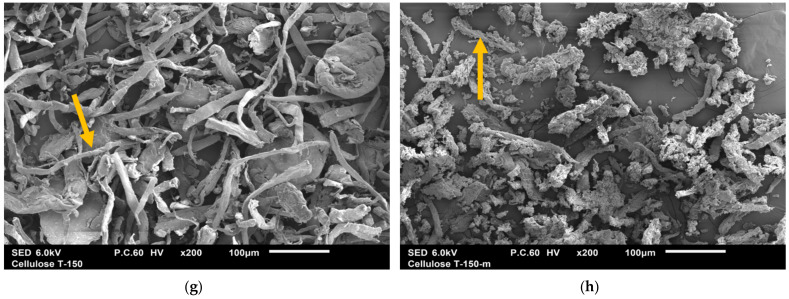
Scanning electron micrographs of cellulose fibers. Orange arrows point on the cellulose fibers, where surface morphology is especially noticeable. Working distance 11 mm, objective aperture (OA) 1. (**a**) Sigmacell not modified, magnification 600×; (**b**) Sigmacell modified, magnification 600×; (**c**) T-40 not modified, magnification 200×; (**d**) T-40 modified, magnification 200×; (**e**) T-90 not modified, magnification 200×; (**f**) T-90 modified, magnification 200×; (**g**) T-150 not modified, magnification 200×; (**h**) T-150 modified, magnification 200×.

Another proof of the successful CNF modification was provided by SEM, Figure 6. The micrographs show the surface morphology of cellulose fibers before and after the modification reaction. Dramatic changes were observed after the incorporation of 4-VBC. The surface modification with vinylbenzyl units disrupts the hydrogen bonding between the individual cellulose fibers causing the roughness. Before the modification, the fibers have smooth surface, but after the modification, they became significantly more porous and hairier, losing their initial surface smoothness (Figure 6a,b, Sigmacell). It should be noted, however, that the overall size and shape of the CNFs were still preserved. The same changes were seen in the surface morphology for all types of Technocell fibers (Figure 6c–h). In these micrographs, one could also notice the difference in sizes and shapes of cellulose fibers. The CNFs of T-90 have the most uniform shape and T-150 the least uniform. 

Disruption in the cellulose fiber structure after the chemical modification has been also previously reported [35,36]. An increase in roughness of the surface is proven to improve the adhesion of cellulose fibers to the polymer matrix due to an increase in the surface area for mechanical interlocking [25,37].

**Figure 7:**
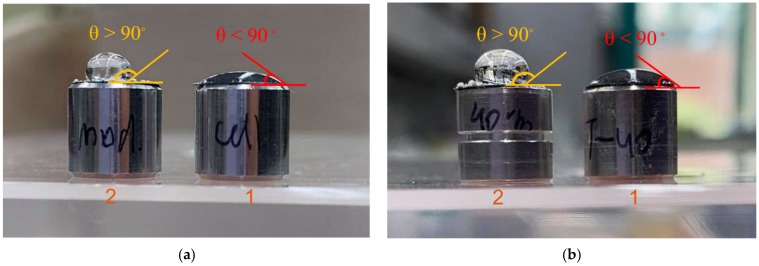

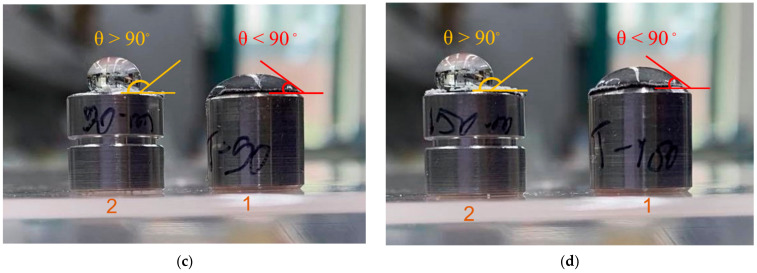
Surface wettability test of not modified (1) and modified (2) cellulose fibers: (**a**) Sigmacell; (**b**) T-40; (**c**) T-90; (**d**) T-150.

The change of surface hydrophilicity of the cellulose fibers could be used to confirm the incorporation of the hydrophobic St moiety and was monitored by contact angle measurement. The images in Figure 7a–d show that all CNFs initially had more hydrophilic surface (‘1’ in Figure 7a–d, θ < 90°), which became significantly more hydrophobic after the 4-VBC modification (‘2’ in Figure 7a–d, θ > 90°). Such a noticeable change in the hydrophobicity of cellulose fibers after the modification reaction is very common, as there is an alteration in the surface properties [20,21,36]. 

### 3.3. Preparation and Characterization of PSt Nanocomposites

PSt-based nanocomposites were synthesized by bulk radical copolymerization of St with cellobiose-m, cellotriose-m and CNF-m. The component weight ratio for all three networks was 1/100/0.5 (cellu-mer/St/AIBN), unless indicated otherwise. After the polymerization, the resulting solids were Soxhlet extracted for four consecutive days with hot chloroform to remove the unreacted St and the non-crosslinked PSt. The recovered polymers were analyzed by SEC every 24 h (Figure S10, Table S1). Figure S11 shows the SEC chromatograms of PSt extracted from cellobiose-m, cellotriose-m and Sigmacell-m composites after the first 24 h. The relatively broad monomodal peaks at retention volume (RV) around 20 mL have similar shape with molecular masses between 50 and 70.5 kDa (Table 3), indicating that all three systems with three different cellu-mers experienced approximately the same polymerization conditions during the chain propagation stage. Notably, the molecular mass of extracted polymers increased with the extraction time, Table 3. It is logical to assume that shorter polymer chains dissolved faster and were preferably extracted. Then, as the gels continued to swell, the pores continued to expand, and so longer polymer chains dissolved and migrated out of the networks. Extractions of cellobiose-m and cellotriose-m nanocomposites also yield low molecular mass fractions, which could possibly contain short-grafted cellu-mers (Figure S10, Table S1 and Table 3). PSt formed in the presence of non-modified fillers was also analyzed (Figure S12 and Table 3). Interestingly, with the increase of cellulose ratio in networks (5 and 10 w%), the length of extracted PSt chain decreases. Figures S13 and S14 show the SEC chromatograms of extracted PSt from those networks. 

The removal of non-crosslinked PSt practically yielded networks consisting of reactive cellu-mers interconnected with PSt segments. Their swelling degree (SD) is an indication of the crosslinking density and could be used as qualitative estimate for the degree of substitution (DS) and the accessibility of the St moieties along the carbohydrate chains. The SD of cellotriose-m gel is higher than that of cellobiose-m (5100% vs. 3800%, respectively), Figure S15. This should be expected, since the average DS for cellotriose-m is lower than that of cellobiose-m (3.03 vs. 3.4, respectively). In other words, there are 1.01 modified groups per 1 anhydroglucose unit in cellotriose-m, and 1.7 modified groups in cellobiose-m. Since both of them are relatively small structures the accessibility of their polymerizable groups did not play a role and therefore the SD (i.e., density of crosslinking) depended solely on the number of those polymerizable groups per molecule. In addition, as modified cellotriose had less polymerizable units, styrene molecules had an opportunity to form longer intercrosslink segments, therefore increasing the swelling degree. Both cellobiose-m and cellotriose-m networks showed remarkable stability and robustness as evidenced by multiple swelling/deswelling sequences, Figure S16.

**Figure 8:**
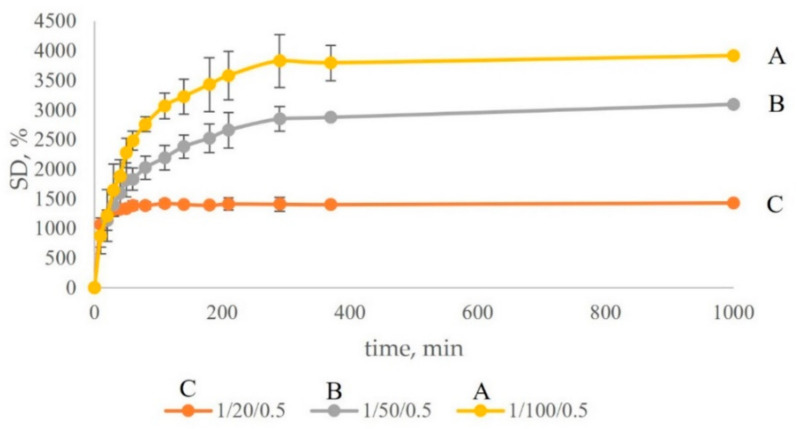
Swelling degree (SD, %) of the cellobiose-m gels with different components ratio of cellobiose-m/styrene/AIBN. (**A**) 1/100/0.5; (**B**) 1/50/0.5; (**C**) 1/20/0.5.

To investigate how the swelling capability depends on the amount of St added, three types of gels were prepared using the same amount and type of crosslinker (cellobiose-m with DS 3.0), the same amount of initiator (AIBN), but different St amounts. The weight ratios cellobiose-m/styrene/AIBN (1/100/0.5; 1/50/0.5; 1/20/0.5) were chosen with sufficient St amount difference to achieve observable SD change. Table 4 and Figure 8 show the change in SD with the composition change. With the decrease of St content, the SD decreases, because the diminishing supply of St monomer leads to the formation of shorter interlink segments in the network. The deswelling experiments show the same tendency, Figure S17. The amount of extracted St (Table 4) indicates that there is a competition between the two processes, crosslinking and homopolymerization, the latter progressively favored with increase in the available amount of St molecules. The information from this experiment provides an important insight for the mechanism that distinguishes this strategy from all other methods for composite production. It essentially yields composites in the form of semi-interpenetrating networks, were the bulk of the polymer formed in situ is fortified by a compatible interdispersed cellu-mer network made at the same time.

Sigmacell-based gels express the highest SD (5600%). Gels, made with Technocell-m fibers swell in the following order T-150 (2200%) > T-90 (1750%) > T-40 (900%), Figure S18. The opacity of cellulose-based samples can be seen in Figure S19.

**Figure 9:**
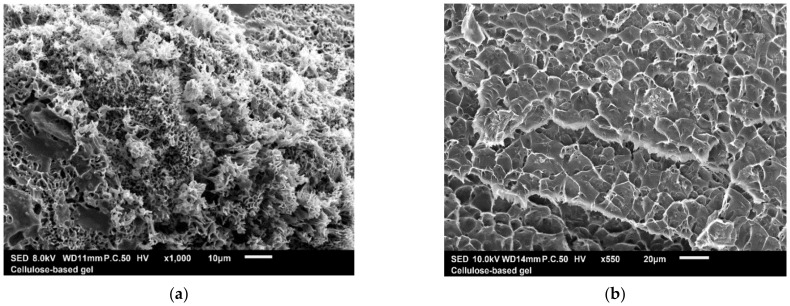
Scanning electron micrographs of PSt composites formed with Sigmacell-m: (**a**) Sample swollen in chloroform and then cryo-fractured. Working distance 11 mm, OA 1, magnification 1000×; (**b**) Sample cryo-fractured in dry state. Working distance 14 mm, OA 1, magnification 550×.

The surface morphology of the PSt gel synthesized with Sigmacell cellu-mer was also studied by SEM using two different protocols. The first one analyzed a nanocomposite specimen swollen in chloroform, frozen in liquid nitrogen, fractured and carbon coated. During the carbon coating in the high vacuum chamber the specimen visibly collapsed. That is why the pores seen in the image might be smaller in size than those in the solvent-swollen sample (Figure 9a). In the second analysis a dry specimen was cryo-fractured and the exposed area in the bulk of the sample showed and interesting lamellar pattern (Figure 9b). 

**Figure 10:**
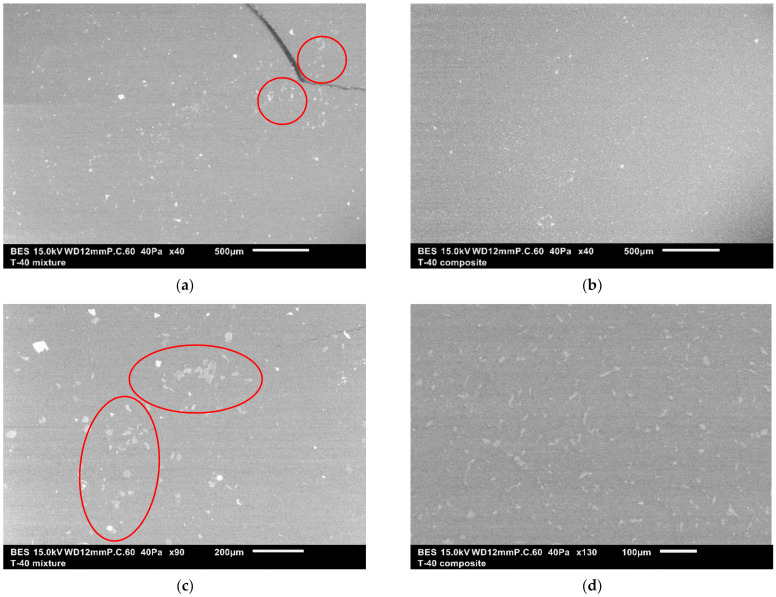
Surface morphology of a T-40 mixture ((**a**,**c**), magnification 40×; and 90×; respectively) and T-40-m composite specimen ((**b**,**d**), magnification 40×; and 90×, respectively). Working distance 25 mm, OA 1, AV 10 kV, P.C. 50.

To assign the white edges of those microflakes as CNF-m would have been purely speculative and therefore a different preparation technique was used to trace those cellulose fibrils in the bulk of the PSt matrix. A composite mold was microtomed and the surface was then analyzed by SEM. Figure 10a,c shows the distribution of the cellulose fibers in the polymer matrix when they were not St-modified. Large agglomerations can be seen (marked with red circles). Figure 10b,d shows a composite material prepared with modified cellulose fibers. Markedly this image showcases the uniform distribution of CNFs throughout the bulk of the PSt, with no large CNF-m agglomerates noticeable. 

**Figure 11:**
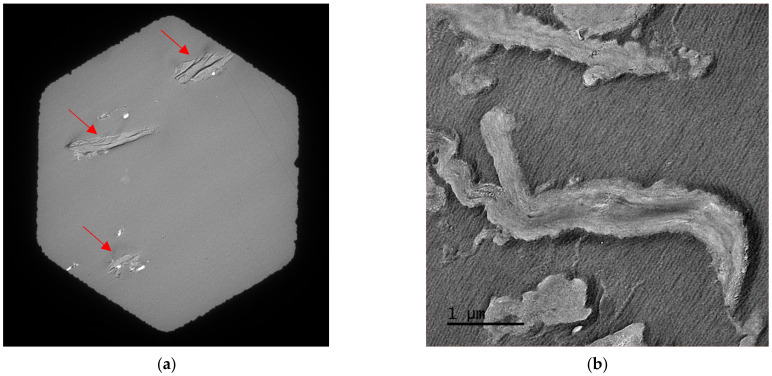
Transmission electron micrographs of Sigmacell-based semi-IPN, stained with 2% uranyl acetate: (**a**) Magnification 800×; (**b**) Magnification 6000×. Red arrows mark visible cellulose fibers.

Thin slices from the same specimen produced by the microtome provided a more detailed insight into the cellulose fibers distribution in the polymer matrix after TEM analysis (Figure 11). The red arrows point towards visible cellulose fibers. It should be emphasized that those fibers are not bundled together, but are distributed as single entities with preserved inner structure, each one surrounded by the PSt matrix (see also Figure 6 for comparison). An image of the pure PSt matrix can be found in the supporting information (Figure S20).

### 3.4. Thermal and Mechanical Analysis of PSt Nanocomposites

The thermal analysis of the synthesized materials was performed using differential scanning calorimetry (DSC). Table 5 contains values of T_g_ and ΔC_p_ obtained from curves that are represented on Figure S21. In case of cellobiose-m specimens, the T_g_ of the extracted linear PSt has the lowest value, but the ΔC_p_ value is the largest. In the case of composite material (both extracted and not extracted specimens), T_g_ increased significantly, while ΔC_p_ declined. Typically, smaller ΔC_p_ is characteristic for less mobile polymer chains. The increase in T_g_, coupled with the lowering of the heat capacity jump in crosslinked samples indicates the onset of polymer immobilization [38]. The same pattern in T_g_ and ΔC_p_ values was observed for cellotriose-m and Sigmacell-m samples. Interestingly, T_g_ for all extracted networks is around 109 °C. This indicates that all three introduced nanofillers have a similar effect on the thermal behavior of the composite, and it is significantly improved compared to both pure PSt and St/cellulose mixtures. Noticeably, with the increase in the nanofiller content to 5 and 10 w%, no significant change in T_g_ values was observed. Two conclusions could be made based on these findings: (1) There is an optimal threshold for favorable filler content above which the additional quantities do not affect the onset of backbone motion in the composite matrix and (2) even at higher cellu-mer concentration an agglomeration did not occur. Typically, in composite materials with uneven blending, the immiscible components form separate domains with an interphase region, and up to three T_g_s could be observed [39]. Importantly, all tested networks showed a single glass transition (Figure S21) meaning that cellu-mer crosslinks were spread evenly across the material.

**Figure 12:**
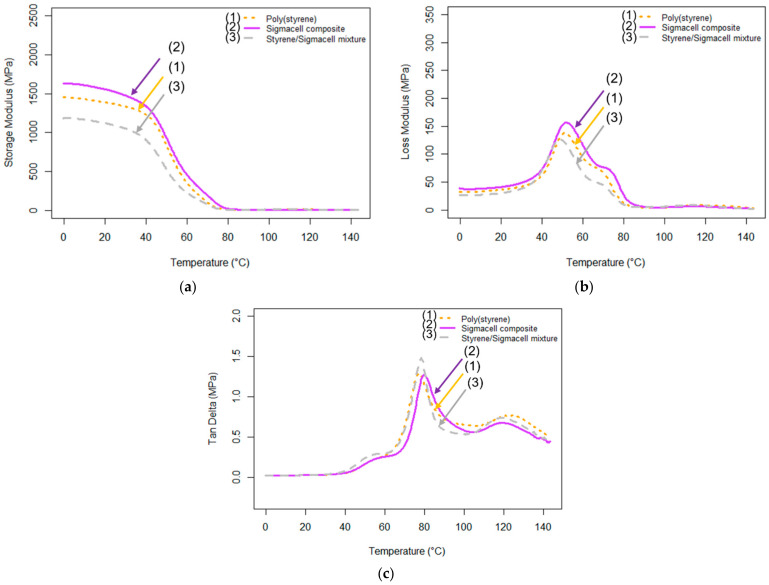
DMA thermograms of tested samples: (**a**) Storage modulus E′, (**b**) Loss modulus E″, (**c**) Tan δ. (1) PSt, (2) composite prepared with Sigmacell-m, (3) physical Sigmacell/PSt mixture.

Dynamic mechanical analysis (DMA) is commonly used to evaluate the rheological characteristics of the materials [40]. It provides information on storage modulus (E′), loss modulus (E”) and Tan δ. E′ represents the elastic component of material behavior and is related to the energy storage in a cycle of deformation. E” represents the viscous component of material behavior and is indirectly proportional to the energy loss as heat in a cycle of deformation. Improvements in the moduli are highly desirable for any new composites, as they reduce the quantities of a material needed to achieve necessary mechanical characteristics. The incorporation of cellulose-m fibers into the matrix was expected to improve the mechanical properties of the resulting s-IPN over the pure matrix. Figure 12 shows E′, E″ and Tan δ of a Sigmacell-m composite with 1 w% cellu-mer. Each graph contains the performance of three samples: Linear PSt (1), semi-IPN composite with CNFs-m (2) and mixture of non-modified CNFs/PSt (3). The storage modulus values at 25 °C for the composite are higher compared to those of PSt and PSt/Sigmacell mixture. This is the result of the rigid filler phase stiffening the bulk material. Sigmacell-m composites also show an increase in the storage modulus, Figure 12b. The damping behavior of the material can be evaluated by the Tan δ of the material. Damping is associated with the reduction in internal friction within the material. Materials with good damping characteristics are extensively used in the aircraft and automobile industries for the reduction of noise and vibration [41]. Addition of a filler compatible to a polymer matrix would induce a restriction in polymer chain mobility and would reduce and broaden the Tan δ peak. Indeed, with the samples analyzed (Figure 12c), the height of dumping peak for the Sigmacell-based composite decreased with the covalent incorporation of cellu-mers. A shift to higher temperatures was observed, as well. 

**Figure 13:**
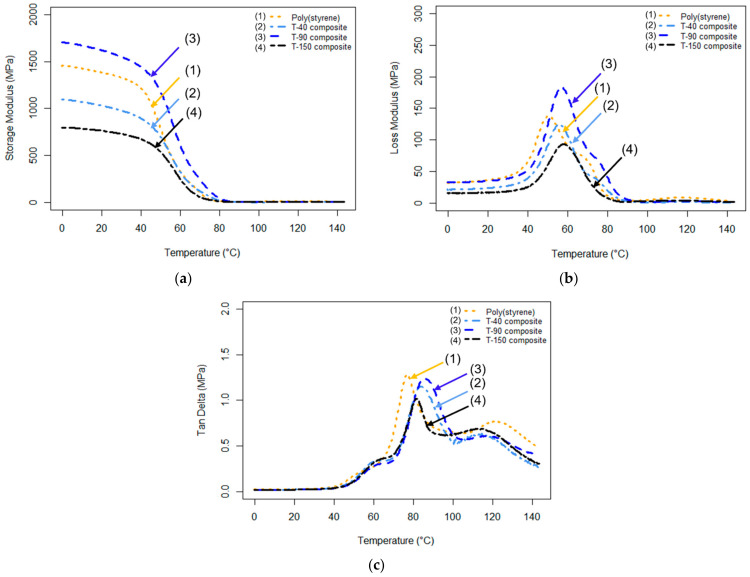
DMA thermograms of tested samples. (**a**) Storage modulus E′ of poly(styrene), (**b**) Loss modulus E″, (**c**) Tan δ of (1) poly(styrene), (2) T-40 composite, (3) T-90 composite and (4) T-150 composite.

Figure 13 shows DMA results of semi-IPNs prepared with Technocell-m cellu-mers at 1 w% content.

The storage modulus (E′) at 25 °C, loss modulus (E″) and Tan δ are summarized in Table 6. At room temperature, composites prepared with Sigmacell and T-90 cellu-mers show storage modulus and loss modulus that are higher than linear PSt and their corresponding physical mixtures. Notably, all physical mixtures have E′ lower than of linear PSt. Tan δ of T-40-m and T-90-m composites is wider than PSt and shifted towards higher temperatures, in distinction to the PSt composites based on T-150-m. The results obtained indicate that Sigmacell-m and T-90-m materials have the most promising mechanical properties of the four composites tested with E′–E″ improvement factors 13–14% and 17–33%, respectively. This could be related to the size distribution of cellulose particles, Sigmacell and T-90 being the most uniform CNFs in size. 

## 4. Conclusions

The results obtained show that polymer composites could be successfully formed by a novel strategy based on the copolymerization of a monomer and complementary modified cellulosic macromonomer. In a proof-of-concept attempt 4-methylstyrene moieties were reproducibly attached to cellulose fibrils, increasing their hydrophobicity and transforming them into multifunctional macromonomers. The reactive cellulose derivatives—cellu-mers—were copolymerized with St through at least two parallel processes running simultaneously: St homopolymerization (minor) and St/cellu-mer crosslinking copolymerization (dominant). The net result of these processes was the formation of composites as semi-IPNs consisting of linear PSt intertwined in a PSt/cellu-mer network. The hypothetical formation of cellu-mer homopolymers or cellu-mer(PSt)n stars could not be detected by the spectroscopic and chromatographic techniques employed to characterize the copolymerization mixtures. Electron microscopy observations indicated that the cellulose-based reactive crosslinking units were uniformly dispersed in the composite without any large agglomerations. The networks formed showed good swelling/reswelling properties in chloroform that depend on the amount of St available at the crosslinking stage. Several of the cellu-mer-containing PSt composites exhibited improved thermal and mechanical properties compared to the physical mixtures of unmodified cellobiose, cellotriose, Sigmacell and Technocell at the same concentration and linear PSt of similar molecular mass. The results obtained confirm the suggested advantages of the cellu-mer approach to polymer composites in avoiding the cellulose fragments agglomeration within intrinsically incompatible polymer matrix (PSt). 

## Figures and Tables

**Table 1:** Molecular mass characteristics of cellobiose-m depending on temperature.

4-VBC addition to cellobiose solution at RT		
M_n_	M_w_	Ð
985	1025	1.04
4-VBC addition to hot cellobiose solution		
M_n_	M_w_	Ð
1151	1174	1.02

M_n_—number average molecular mass; M_w_—weight average molecular mass; Ð –dispersity index. Calculated using MALDI-TOF spectra and Equations (1) and (2).

**Table 2:** Particle size distribution of cellulose fibers (provided by the manufacturers).

Cellulose Type	Particle Size Distribution (Long Axis), % Passing through Sieves		
	32 μm	50 μm	90 μm
Technocell 40	>97	>99	
Technocell 90	>50	>80	>99
Technocell 150	>35		>85
Sigmacell	>99		

**Table 3:** Molecular mass and dispersity index (Ð) of non-crosslinked PSt fractions extracted from cellobiose-m (CB-PSt), cellotriose-m (CTR-PSt), cellulose-m (CELL-PSt) gels and of PSt polymerized in a presence of a non-modified carbohydrate filler.

PSt Network	M_n_, kDa	Ð
**CB-PSt** Day 1	70,500	3.04
**CTR-PSt** Day 1		
	55,500 + 386	3.55 + 1.07
**CELL-PSt**, 1 w% Day 1	50,000	5.01
**CELL-PSt**, 5 w% Day 1	47,800	6.10
**CELL-PSt**, 10 w% Day 1	32,500	4.24
**Mixtures** PSt/CB	60,300	2.10
PSt/CTR	76,000	1.60
PSt/CELL	51,500	2.11

**Table 4:** SD changes depending on cellobiose-m/styrene/AIBN weight ratio.

Reagents Ratio	PSt Extracted, w%	SD, w%
1/100/0.5	40.5	3914 ± 300
1/50/0.5	37.5	3093 ± 152
1/20/0.5	31.2	1434 ± 138

**Table 5:** Glass transition temperatures (Tg) and heat capacity jump (ΔCp) for samples obtained from DSC. “PSt extracted” shows how much weight % of non-crosslinked PSt was extracted from the sample during the extraction process.

Composite/Specimen	Material	T_g_, °C	ΔC_p_, J/g°C	PSt Extracted, w%
Cellobiose-m	PSt/CB-m composite not extracted	108.10	0.608540925	
	PSt/CB-m composite extracted	108.81	0.862481752	40.50
Cellotriose-m	PSt/CTR-m composite not extracted	87.48	0.602774566	
	PSt/CTR-m composite extracted	109.07	0.877153558	41.01
Sigmacell-m	PSt/CELL-m composite 1 w% not extracted	90.84	0.736936937	
	PSt/CELL-m composite 1 w% extracted	109.13	0.640086831	36.70
	PSt/CELL-m composite 5 w% not extracted	83.83	1.537828371	
	PSt/CELL-m composite 5 w% extracted	105.62	1.158588235	40.72
	PSt/CELL-m composite 10 w% not extracted	81.76	1.584148728	
	PSt/CELL-m composite 10 w% extracted	108.02	0.757317073	55.24
Linear PSt	Synthesized at the same conditions, 67.5 kDa	94.26	0.883978638	
	PSt standard 130 kDa	109.20	1.346746988	

**Table 6:** Storage modulus (E′) at 25℃, loss modulus (E″) and Tan δ for composites and physical mixtures at 1 w% cellu-mer content.

Material	E′, MPa	E″, MPa	Tan δ
PSt	1362	138	1.28
Sigmacell-m composite	1537	157	1.27
T-40-m composite	1008	124	1.15
T-90-m composite	1588	184	1.24
T-150-m composite	748	93	1.00
Sigmacell mixture	1090	126	1.48
T-40 mixture	1501	170	1.41
T-90 mixture	1040	134	1.16
T-150 mixture	1111	134	1.02

## Data Availability

All results obtained in this study are included in the paper. Any questions or requests can be referred to the corresponding author.

## Supplementary Materials

The following supporting information can be downloaded at: https://www.mdpi.com/article/10.3390/polym14091670/s1, Figure S1: Schematic representation of the cellulose grafting approaches: ‘‘grafting-to’’ and ‘‘grafting-from’’; Figure S2. Calibration curve that was used to calculate the degree of substitution of modified compounds; Figure S3. MALDI-TOF spectra of cellobiose-m obtained with various molar equivalents of 4-VBC at 25 °C for 22 h; Figure S4. MALDI-TOF spectra of cellobiose-m with added 4-VBC; Figure S5. UV-Vis absorption spectra of cellobiose-m; Figure S6. Degree of substitution of cellobiose-m at various 4-VBC molar equivalents; Figure S7. MALDI-TOF spectra of cellotriose modification; Figure S8. UV-Vis absorption spectrum of modified cellotriose; Figure S9. Calculated amount of the number of 4-VBC groups attached to 1g of cellulose using four different types of cellulose; Figure S10. SEC chromatograms of PSt samples extracted from cellobiose-m (a), cellotriose-m (b) and Sigmacell-m (c) based semi-IPNs.; Figure S11. SEC chromatograms of poly(styrene) samples extracted after 24 h from cellobiose-m, cellotriose-m, and Sigmacell-m based networks; Figure S12. SEC chromatorgrams of PSt formed in the presence of non-modified fillers; Figure S13. SEC chromatograms of PSt samples extracted from Sigmacell-m based semi-IPN with 5 w% of a filler; Figure S14. SEC chromatograms of PSt samples extracted from Sigmacell-m based semi-IPN with 10 w% of a filler; Figure S15. Swelling degree of PSt gels containing multi-functional cellobiose-m and cellotriose-m as crosslinks; Figure S16. Swelling/deswelling capabilities of networks with cellobiose-m and cellotriose-m; Figure S17. Deswelling of the gel with different components ratio; Figure S18. SD of the CNF-m gels formed with the same reagents’ ratio; Figure S19. Opacity of PSt/cellulose composites; Figure S20. Transmission electron micrograph of PSt, stained with 2% uranyl acetate, magnification 8000×; Figure S21. DSC thermograms of synthesized materials; Table S1: Molecular mass and dispersity index (Ð) of non-crosslinked PSt extracted from cellobiose-m (CB-PSt), cellotriose-m (CTR-PSt), Sigmacell-m (CELL-PSt) gels and of PSt polymerized in a presence of a non-modified carbohydrate filler.
