# Supplementary material for: Reactive Cellu-mers—A Novel Approach to Improved Cellulose/Polymer Composites
Source: Polymers (Basel). 2022 Apr 20;14(9):1670. doi: 10.3390/polym14091670 (PMC9103100; doi:10.3390/polym14091670)
Supplement: Supplementary file 1 [file polymers-14-01670-s001.zip › polymers-1690664-supplementary.pdf]

Supporting Information

Reactive ‘Cellu-mers’ – a Novel Approach to Improved Cellulose/Polymer Composites

Dariya Getya,[1,2] Ivan Gitsov[1,2,3]

[1] Department of Chemistry, State University of New York – ESF, Syracuse, NY 13210
[2] The Michael M. Szwarc Polymer Research Institute, Syracuse, NY 13210
[3] The BioInspired Institute, Syracuse University, Syracuse, NY 13244, USA

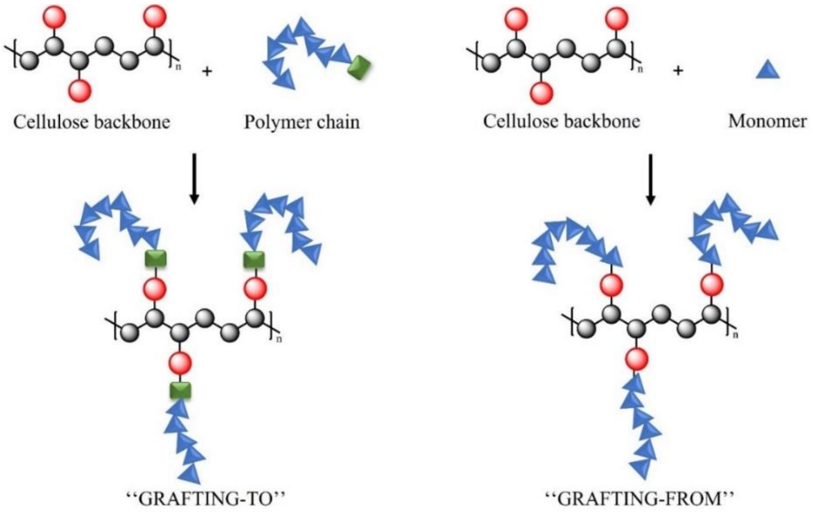

Figure S1. Schematic representation of the cellulose grafting approaches: ''grafting-to'' and ''grafting-from''. Red circles represent available reactive groups on cellulose chain, and green squares represent the reactive polymer chain end.

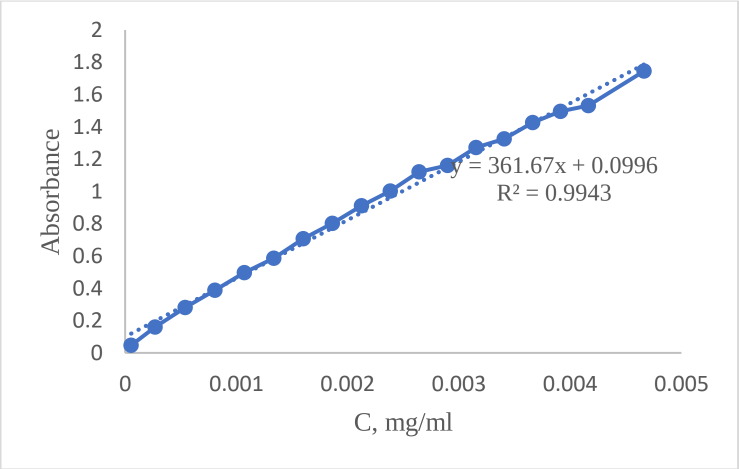

Figure S2. Calibration curve used to calculate the degree of substitution of St-modified compounds. 4-vinylbenzyl chloride UV-VIS absorbance at 265 nm was used.

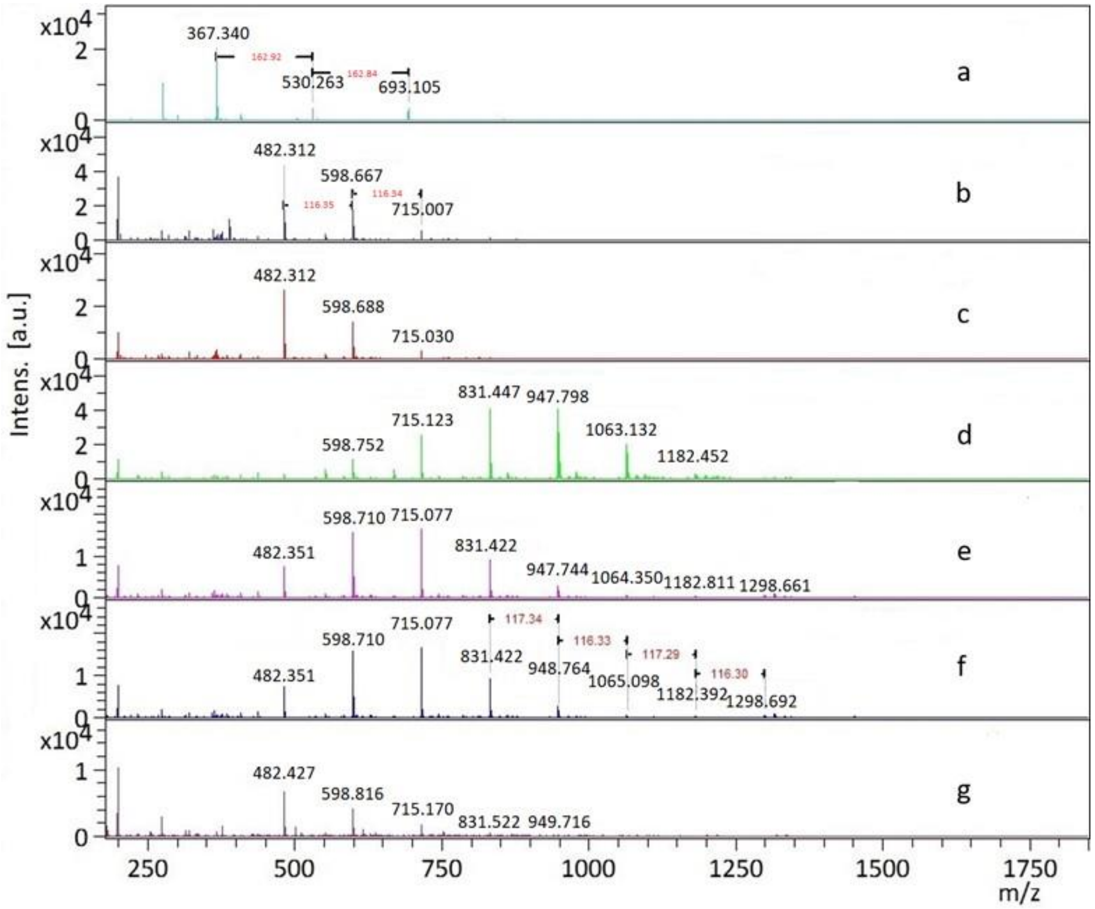

Figure S3. MALDI-TOF spectra of cellobiose-m obtained with various molar equivalents of 4-VBC at 25℃ for 22 h. a) starting cellobiose; b-g) with added 1, 2, 4, 6, 8 and 30 equivalents of 4-VBC, respectively. DHB was used as a matrix.

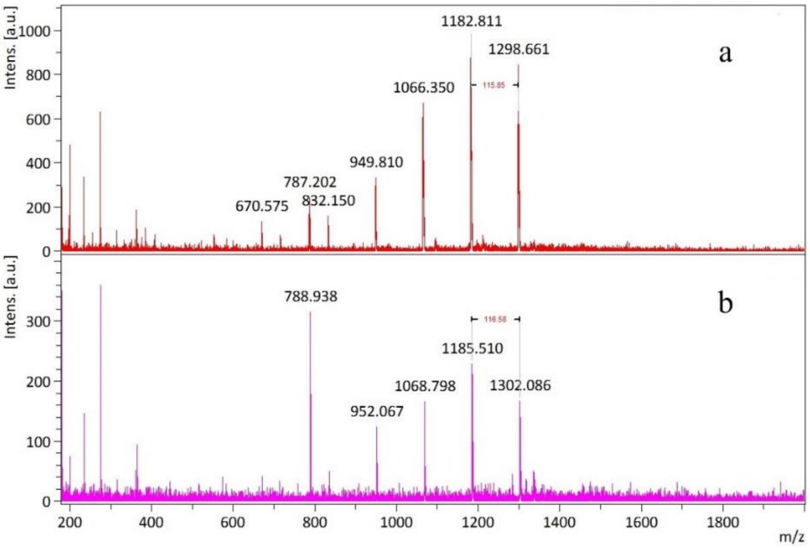

Figure S4. MALDI-TOF spectra of cellobiose-m with 4-VBC added at a) room temperature; b) 100℃. Reaction time 22 h, DHB used as a matrix.

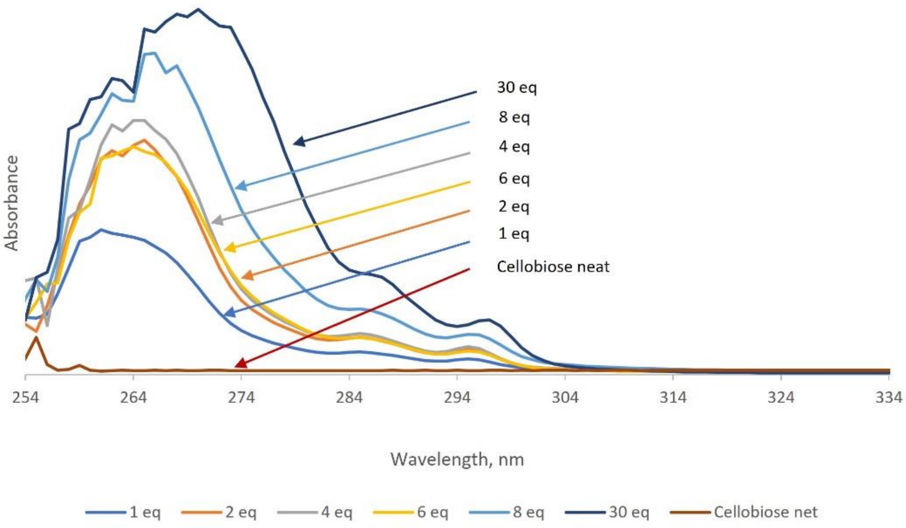

Figure S5. UV-Vis absorption spectra of cellobiose-m. Absorbance is increasing with the increase in number of molar equivalents of the 4-vinylbenzyl chloride used for modification:1-6) 1, 2, 4, 6, 8 and 30 equivalents of 4-VBC, respectively; spectrum of pure cellobiose (0 equivalents) is also included.

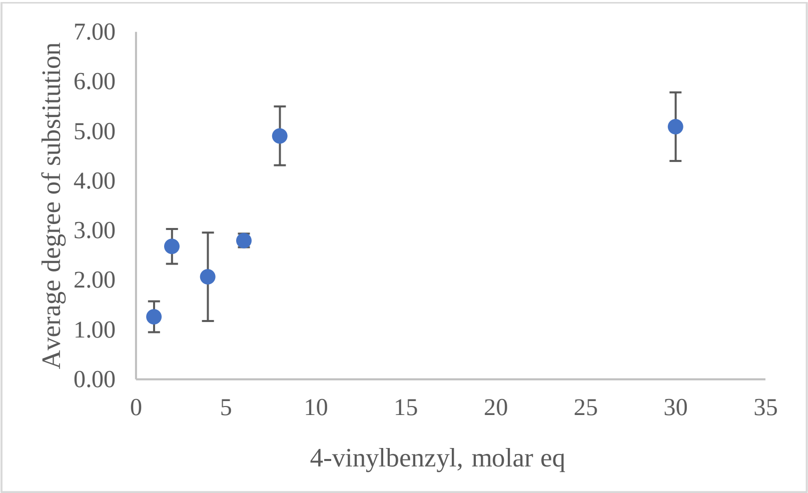

Figure S6. Degree of substitution of cellobiose-m at various 4-VBC molar equivalents. Calculated using the UV calibration curve at 265 nm (Figure S2).

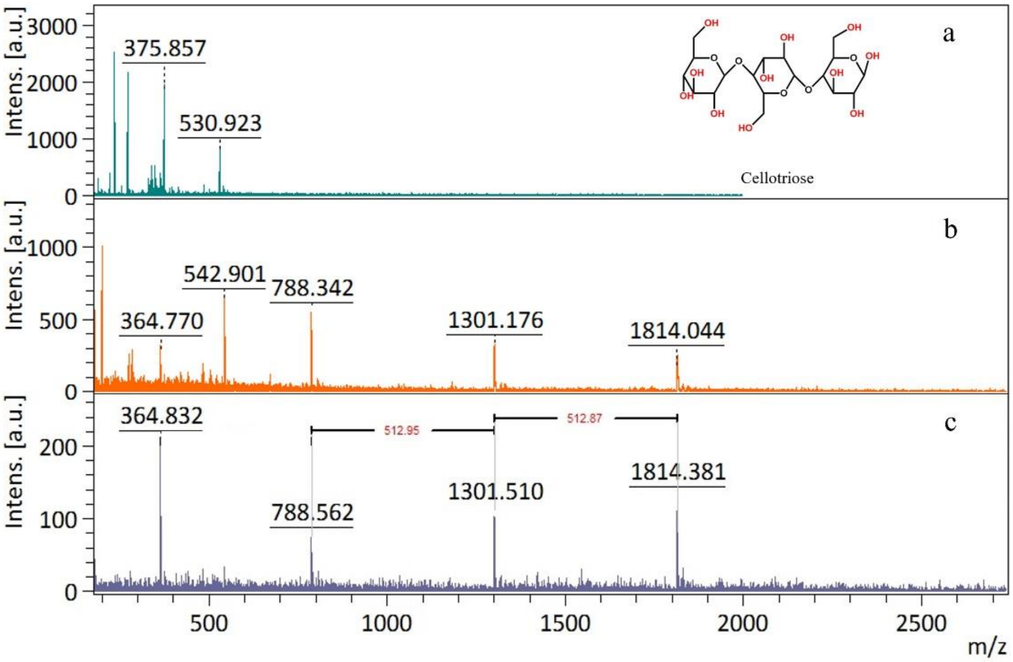

Figure S7. MALDI-TOF spectra of cellotriose modification. a) net cellotriose; b) cellotriose reaction mixture after 16 h of the reaction; c) modified cellotriose after 24 h of the reaction. DHB was used as the matrix.

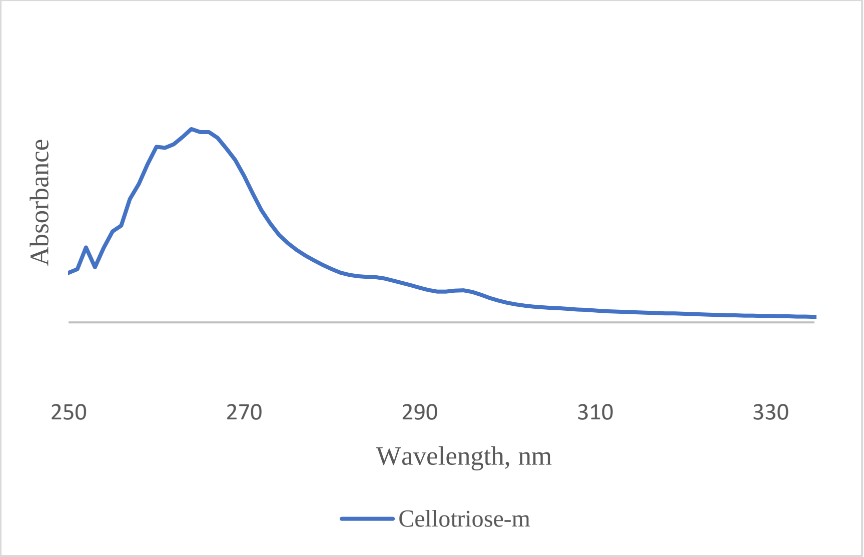

Figure S8. UV-Vis absorption spectrum of modified cellotriose. DS was calculated using the UV calibration curve at 265 nm (Figure S2).

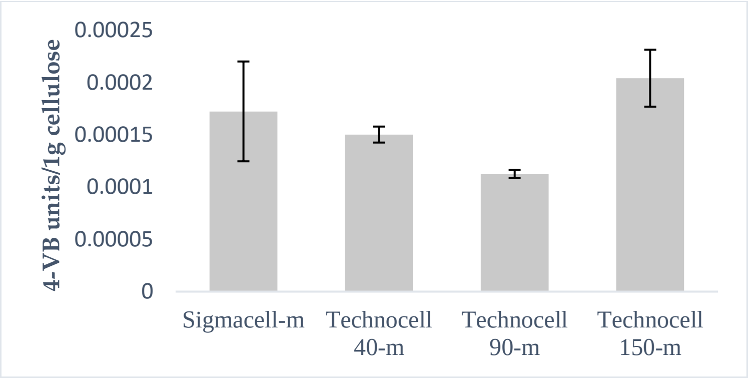

Figure S9. Calculated amount of the number of 4-VBC groups attached to 1g of cellulose using four different types of cellulose. Values were calculated using the calibrated UV-Vis absorbance at 265 nm.

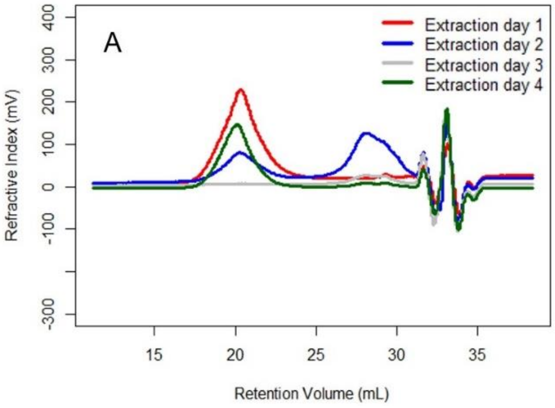

(**a**)

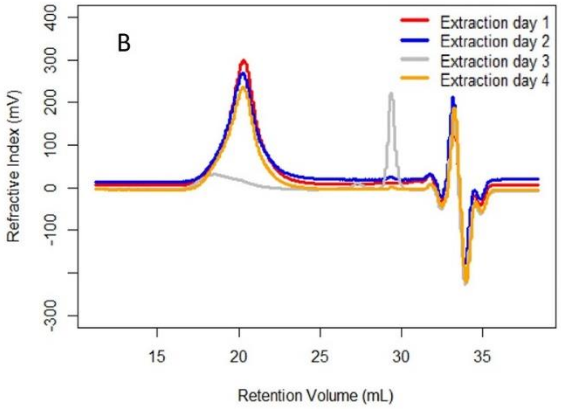

(**b**)

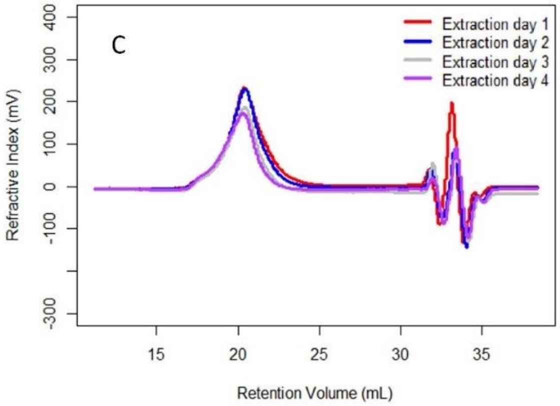

(c)

Figure S10. SEC chromatograms of PSt samples extracted from cellobiose-m (A), cellotriose-m (B) and Sigmacell-m (C) based semi-IPNs.

Table S1. Molecular mass and dispersity index (Đ) of non-crosslinked PSt extracted from cellobiose-m (CB-PSt), cellotriose-m (CTR-PSt), Sigmacell-m (CELL-PSt) gels and of PSt polymerized in a presence of a non-modified carbohydrate filler.

PSt network	Mn, kDa	Đ
CB-PSt		
Day 1	70,500	3.04
Day 2	65,000	3.17
Day 3	155,000	5.58
	1,800	1.04
	360	1.07
Day 4	76,000	2.99
CTR-PSt		
Day 1	55,500	3.55
	386	1.07
Day 2	60,000	3.03
	460	2.56
Day 3	570	1.87
Day 4	81,000	2.65
CELL-PSt, 1w%		
Day 1	50,000	5.01
Day 2	64,000	3.99
Day 3	79,000	3.77
Day 4	98,000	3.49
CELL-PSt, 5w%		
Day 1	47,800	6.1
CELL-PSt, 10w%		
Day 1	32,500	4.24
Mixtures		
PSt/CB	60,300	2.10
PSt/CTR	76,000	1.60
PSt/CELL	51,500	2.10

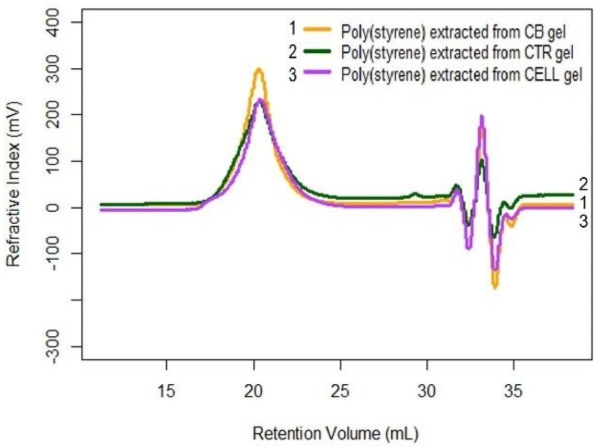

Figure S11. SEC chromatograms of poly(styrene) samples extracted after 24 h from (1) cellobiose-m (CB- gel), (2) cellotriose-m (CTR- gel), and (3) Sigmacell-m (CELL- gel) based networks.

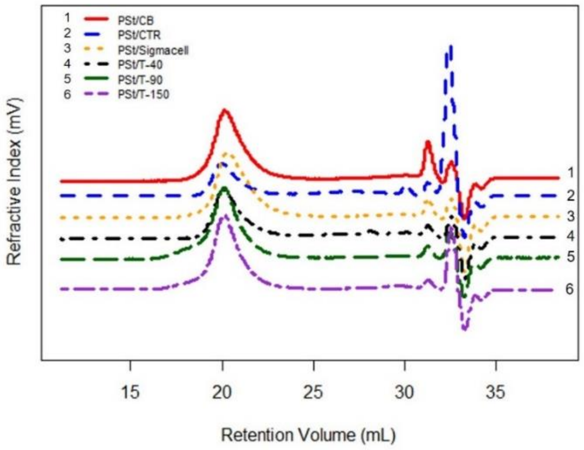

Figure S12. SEC chromatorgrams of PSt formed in the presence of non-modified fillers. 1) PSt formed in the presence of cellobiose; 2) PSt formed in the presence of cellotriose; 3) PSt formed in the presence of Sigmacell cellulose; 4) PSt formed in the presence of T-40 cellulose; 5) PSt formed in the presence of T-90 cellulose; 6) PSt formed in the presence of T-150 cellulose.

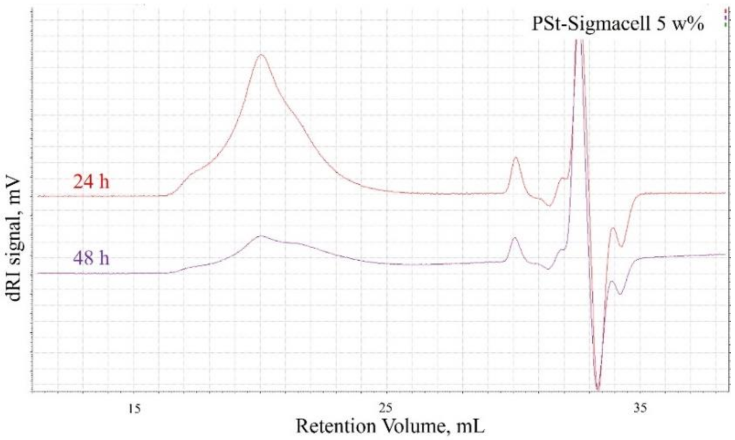

Figure S13. SEC chromatograms of PSt samples extracted from Sigmacell-m based semi-IPN with 5 w% of a filler. Extracted for 24 and 48 hours.

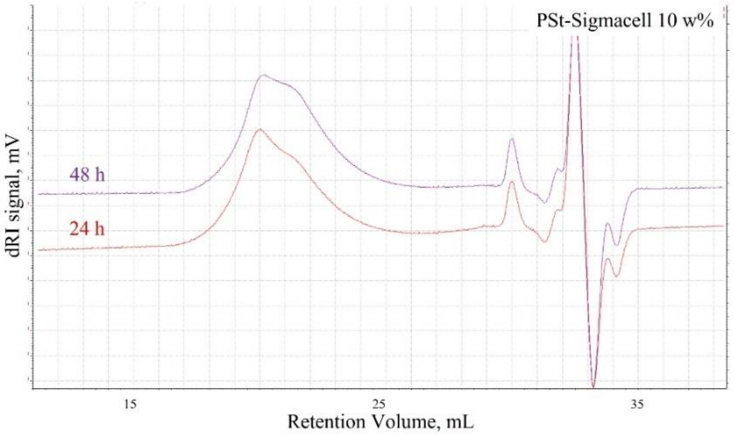

Figure S14. SEC chromatograms of PSt samples extracted from Sigmacell-m based semi-IPN with 10 w% of a filler. Extracted for 24 and 48 hours.

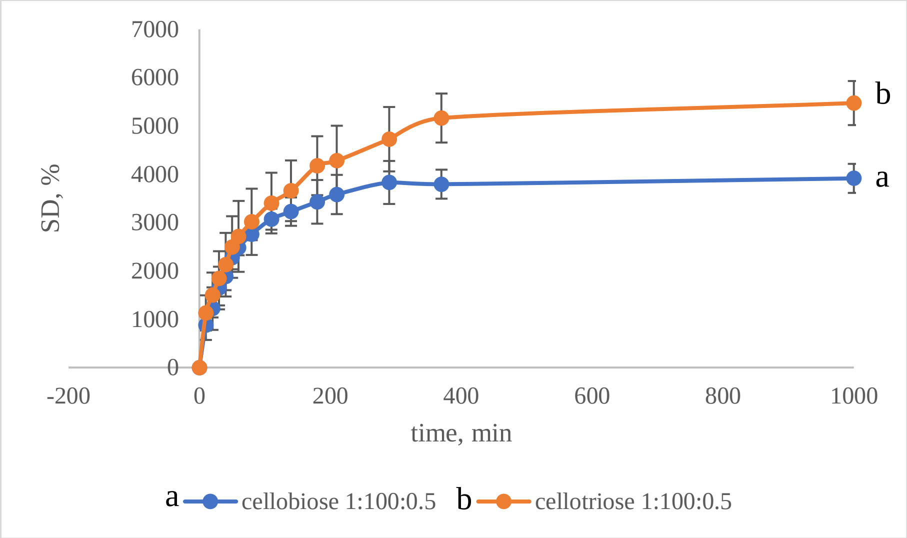

Figure S15. Swelling degree (SD, %) of PSt gels containing multi-functional cellobiose-m (a) and cellotriose-m (b) as crosslinks.

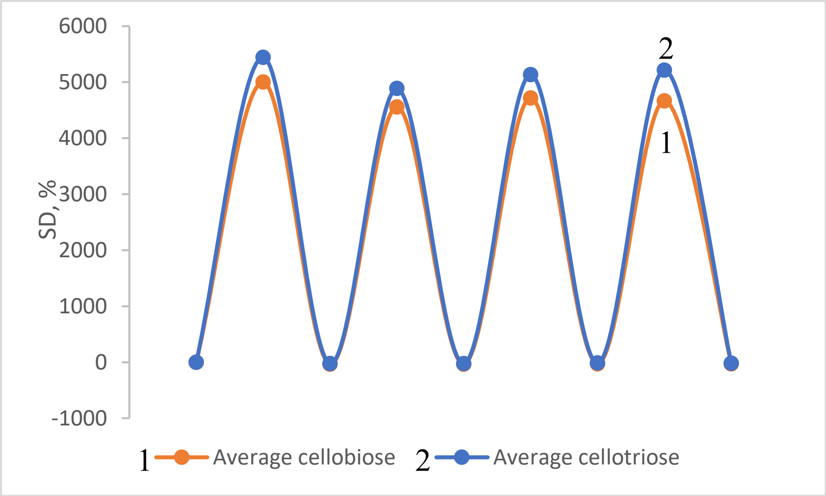

Figure S16. Swelling/deswelling capabilities of networks with cellobiose-m (1) and cellotriose-m (2). Four cycles are shown.

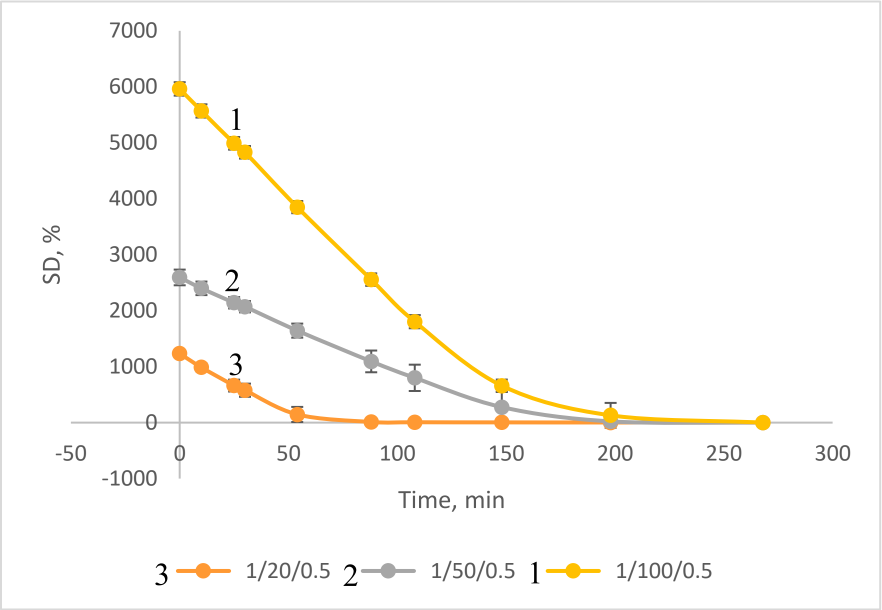

Figure S17. Deswelling of the gel with different components ratio. 1) 1/100/0.5; 2) 1/50/0.5; 3) 1/20/0.5

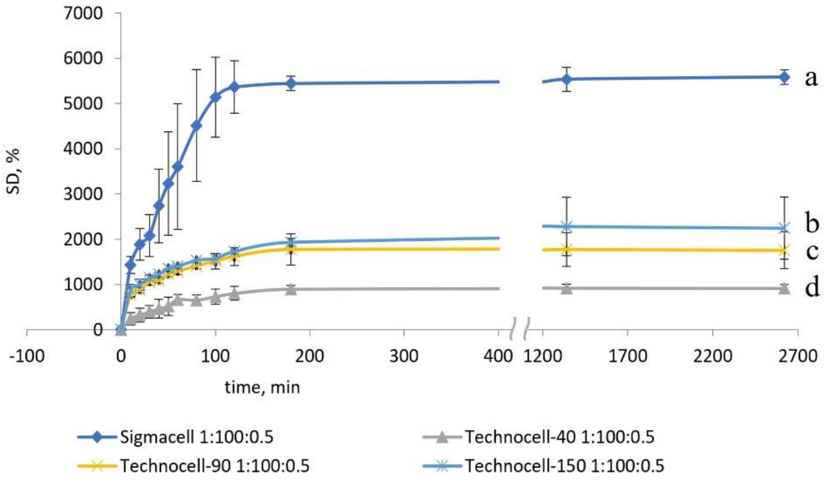

Figure S18. SD of the CNF-m gels formed with the same reagents' ratio. a) Sigmacell 1:100:0.5; b) Technocell-150 1:100:0.5; c) Technocell-90 1:100:0.5; d) Technocell-40 1:100:0.5.

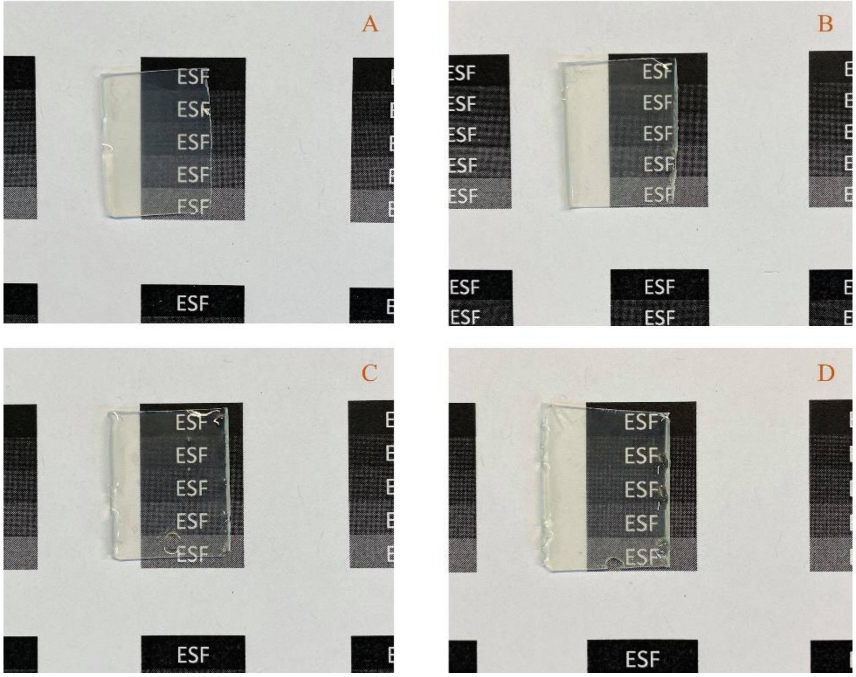

Figure S19. Opacity of PSt/cellulose composites. Material thickness -1.5 mm. A) Sigmacell-m composite, B) T-40-m composite, C) T-90-m composite, D) T-150-m composite.

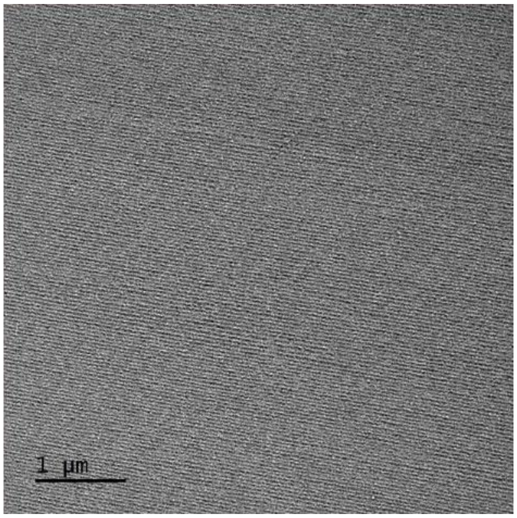

Figure S20. Transmission electron micrograph of PSt, stained with 2% uranyl acetate, magnification 8000×.

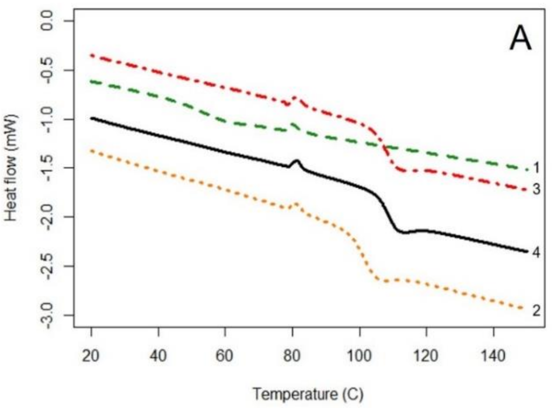

(**a**)

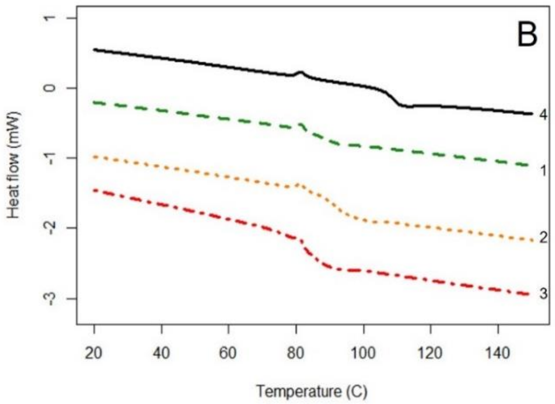

(**b**)

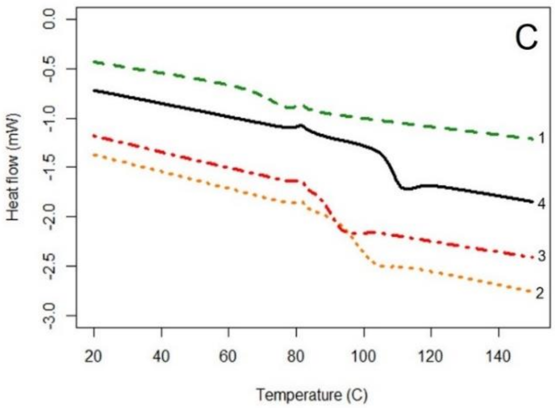

(c)

Figure S21. DSC thermograms of synthesized materials: A) cellobiose-m materials; B) cellotriose-m materials; C) Sigmacell-m materials. Numbers indicate: (1) linear poly(styrene) extracted from the corresponding network, (2) filler/poly(styrene) mixture, (3) not extracted semi-IPN, (4) Extracted network.
